# A Novel *LAS1L* Gene Mutation Associated with Impaired Growth and Developmental Delay and a Review with Previously Reported Cases

**DOI:** 10.3390/genes17060708

**Published:** 2026-06-20

**Authors:** Niusha Mostafavi, Anran Tian, Yuan Gao, Yingying Li, Furong Liang, Cai Zhang, Xiaoping Luo

**Affiliations:** 1 Department of Pediatrics, Tongji Hospital, Tongji Medical College, Huazhong University of Science and Technology, Wuhan 430030, China; niousha.mostafavi@gmail.com (N.M.); anrantian@hust.edu.cn (A.T.); d202382140@hust.edu.cn (Y.G.); lyypediatrician@163.com (Y.L.); lfrdoctor@163.com (F.L.); caizhang@tjh.tjmu.edu.cn (C.Z.); 2 Hubei Key Laboratory of Pediatric Genetic Metabolic and Endocrine Rare Diseases, Wuhan 430030, China; 3 Hubei Provincial Clinical Research Center for Children’s Growth and Development and Metabolic Diseases, Wuhan 430030, China; 4 State Key Laboratory for Diagnosis and Treatment of Severe Zoonotic Infectious Disease, Wuhan 430030, China

**Keywords:** Wilson–Turner syndrome, *LAS1L*, short stature, frameshift mutation, rare genetic diseases, X-linked developmental disorder

## Abstract

Wilson–Turner syndrome (WTS) is an X-linked developmental disorder associated with variants in the *LAS1L* gene, which plays a role in ribosome biogenesis. We report a 6-year-and-5-month-old boy presenting with growth retardation, early developmental delay, and mild scoliosis. Exome sequencing analysis identified a novel hemizygous *LAS1L* frameshift variant, c.2082dup (p.Leu697ProfsTer59), inherited from his asymptomatic mother that was absent from population databases. Functional analysis in HEK-293T cells suggested reduced protein expression with a partial loss of function effect, while structural modeling indicated potential alteration of the C-terminal region. The patient lacked classical WTS features, including craniofacial dysmorphism, truncal obesity, hypogonadism, and neuromuscular involvement. This case expands the phenotypic spectrum of LAS1L-related disorders and highlights the consideration of *LAS1L* variants in children with unexplained growth failure, scoliosis, or developmental delay, even in the absence of classical WTS features.

## 1. Introduction

Wilson–Turner syndrome (WTS; OMIM #309585) is an ultra-rare X-linked recessive disorder, characterized by variable combinations of developmental delay or intellectual disability, speech impairment, hypotonia, distinctive craniofacial features, obesity, gynecomastia, and hypogonadism [1,2,3,4]. It was first described in 1991 in a family with 14 affected males across three successive generations, who presented with X-linked intellectual disability, obesity, gynecomastia, speech difficulties, emotional lability, tapering fingers, and small feet [4]. To date, only two molecularly confirmed families with classic WTS linked to *LAS1L* have been reported. Including these cohorts, a total of five distinct *LAS1L*-associated case groups have been documented prior to the present study [2,3,5,6]. Clinical manifestations vary across individuals and age groups; features such as obesity, gynecomastia, and hypogonadism often do not fully manifest until late childhood or adolescence. Accordingly, the full clinical and molecular spectrum of this disorder remains incompletely defined. *LAS1L* is located at Xq12 and encodes a nucleolar protein essential for ribosome biogenesis. It functions alongside PELP1, TEX10, and WDR18 to facilitate internal transcribed spacer 2 (ITS2) processing during assembly of the 60S ribosomal subunit, as well as interacting with NOL9 to regulate pre-rRNA cleavage. Disruption of LAS1L-mediated ribosome biogenesis has been shown to induce p53-dependent cellular responses in model systems [7,8,9,10].

The five previously reported LAS1L-related case groups include two classic WTS families carrying the variants p.Ala269Gly and p.Arg415Trp, two patients with severe infantile neuromuscular disease harboring c.1430G>A (p.Ser477Asn) and c.846G>C (p.Thr282=), and one individual with an Angelman-like neurodevelopmental phenotype carrying c.1237G>A (p.Gly413Arg) [2,6,11,12]. Reports of the *LAS1L* variants c.1430G>A and c.846G>C describe clinical phenotypes distinct from classical WTS, including severe infantile motor neuron disease, profound hypotonia, progressive motor neuropathy, feeding difficulties, and respiratory compromise [11,12].

These findings highlight the broad phenotypic diversity resulting from *LAS1L* mutations and underscore the need for further investigation into its role in ribosome biogenesis and human developmental disorders. Collectively, prior studies demonstrate that LAS1L-related disorders exhibit a wider phenotypic spectrum than classic WTS alone, although the small number of published cases precludes definitive genotype–phenotype correlations [1,13].

Here, we report a 6-year-and-5-month-old boy presenting with growth retardation, mild developmental delay, and early scoliosis, who carries a novel hemizygous *LAS1L* frameshift variant: c.2082dup, p.Leu697ProfsTer59. We describe the patient’s clinical and genetic features alongside a concise summary of all previously reported LAS1L variants, further expanding the recognized phenotypic spectrum of *LAS1L*-associated disease. This patient represents the sixth documented LAS1L-associated case group and presents a mild–intermediate phenotype within the broad spectrum of LAS1L-related developmental disorders.

## 2. Materials and Methods

### 2.1. Patient Enrollment and Clinical Investigations

A 6-year-and-5-month-old boy was enrolled in this study after obtaining written informed consent from his parents. Comprehensive clinical and laboratory evaluations were conducted, including assessments of growth parameters, skeletal development, and metabolic indicators. Physical examinations and medical history were reviewed to identify possible dysmorphic features, behavioral characteristics, and endocrine abnormalities. All procedures were conducted in accordance with institutional ethical guidelines.

### 2.2. Ethics Statement

This study was reviewed and approved by the Ethics Committee of Tongji Hospital, Tongji Medical College, Huazhong University of Science and Technology (TJ-IRB20201016). All procedures were performed in accordance with the Declaration of Helsinki. Written informed consent was obtained from the parents prior to participation.

### 2.3. Exome Sequencing

Genomic DNA was extracted from peripheral blood samples of the patient and his family using the QIAamp DNA Blood Mini Kit (Qiagen GmbH, Hilden, Germany). DNA libraries were prepared by fragmentation, followed by library construction according to MyGenostics protocols (MyGenostics, Beijing, China). Target enrichment for whole-exome sequencing was performed using the GenCap WES capture kit (MyGenostics, Beijing, China). Sequencing was carried out on an MGI DNBSEQ-T7 platform, with an average sequencing depth of approximately 204.8× and 98.73% of target regions covered at ≥20×. Sequence reads were processed and analyzed using the BWA-GATK pipeline and aligned to the human reference genome (UCSC hg19) (bio-bwa.sourceforge.net; software.broadinstitute.org/gatk/) (accessed on 17 June 2026).

Variant annotation was performed using ANNOVAR (http://annovar.openbioinformatics.org/en/latest/) (accessed on 17 June 2026) and cross-referenced against multiple databases, including the 1000 Genomes Project, dbSNP, Human Gene Mutation Database (HGMD), and MyGenostics local database. Variants were filtered based on allele frequency (excluding variants with a minor allele frequency > 1%), predicted functional impact (including nonsynonymous, frameshift and splice site variants), and consistency with the inheritance pattern. Synonymous variants without predicted functional significance were excluded. Candidate variants were prioritized based on relevance to the patient’s phenotype and the X-linked inheritance model. No alternative candidate variants with a stronger genotype–phenotype correlation were identified.

To validate the *LAS1L* variant identified by next-generation sequencing, the relevant exons and exon–intron boundaries were amplified, purified, and sequenced using an ABI 3730XL sequencer (Applied Biosystems; Thermo Fisher Scientific, Inc., Waltham, MA, USA). Sequence data were analyzed using Mutation Surveyor software (version 4.0.4; SoftGenetics, LLC).

### 2.4. Functional Bioinformatics Assessment of the Identified Variants

Protein multiple sequence alignment, amino acid hydrophobicity, and conservation analyses were performed using DNAMAN version 6.0.3.99 (Lynnon Biosoft, San Ramon, CA, USA). Evolutionary conservation at the affected amino acid position was assessed to determine whether the substitution occurs within a conserved domain of *LAS1L*. Hydrophobicity profiling was used to evaluate potential changes in local physicochemical properties that may affect protein folding and stability, though functional consequences cannot be inferred directly from these analyses.

### 2.5. Plasmid Construction

The wild-type *LAS1L* cDNA was cloned into the pcDNA3.1-Flag-C eukaryotic expression vector. Mutant constructs were generated using the QuikChange site directed mutagenesis kit (Stratagene, La Jolla, CA, USA) with the wild-type plasmid as a template. All plasmids were verified by Sanger sequencing using the primers listed in Supplementary Table S1.

### 2.6. Cell Culture and Transfection

HEK-293T cells were cultured in Dulbecco’s Modified Eagle Medium (DMEM) supplemented with 10% fetal bovine serum and 1% penicillin–streptomycin at 37 °C in a 5% CO_2_ incubator. Cells were plated in 12-well dishes and transfected at 70–80% confluence, with 2 µg of *LAS1L* plasmid DNA and 4 µL of Lipofectamine 3000 (Invitrogen, Carlsbad, CA, USA) per well following the manufacturer’s instructions. A GFP control vector was included in parallel transfections. After 48 h, cells were collected and processed for downstream analyses.

### 2.7. Western Blot

Proteins were collected from transfected HEK-293T cells using RIPA buffer (BOSTER, Wuhan, China). Samples were separated on 10% SDS PAGE and transferred to nitrocellulose membranes. Membranes were blocked for 2 h with 5% skim milk at room temperature and incubated overnight at 4 °C using the primary antibodies mentioned in Supplementary Table S2. Following washes, membranes were incubated with secondary antibodies and signal detection was performed using a ChemiDoc XRS+ system (Bio-Rad Laboratories, Hercules, CA, USA). Band intensity was examined with Image Lab software (version 6.0; Bio-Rad Laboratories, Hercules, CA, USA). Full-length, original, and unprocessed blot images are provided in Supplementary Figure S1.

### 2.8. Literature Search Strategy and Data Analysis

Reports and case series describing *LAS1L* variants were searched in PubMed and HGMD using the terms “*LAS1L*”, “Wilson–Turner syndrome”, and “X-linked intellectual disability”. The search deadline was August 2025. Only studies with molecular confirmation were considered. From eligible publications, patient details, clinical features, and *LAS1L* variants were extracted. These data were used to summarize reported phenotypes and variant characteristics associated with *LAS1L* without implying causality.

## 3. Results

### 3.1. Case Report

#### Clinical Characteristics

**Figure 1:**
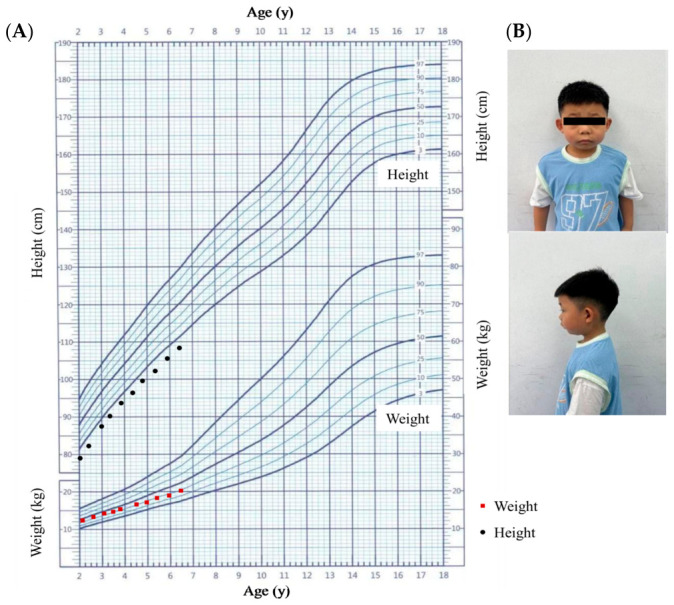
Growth curves and characteristic phenotype of the patient. (**A**) Postnatal growth curve of height and weight of the patient. (**B**) Frontal and lateral photographs of the patient at 6 years and 5 months, showing an appearance with a short forehead, small jaw, large ears and subtle craniofacial features.

A 6-year-and-5-month-old Chinese Han boy was admitted to our hospital for evaluation of growth retardation, as noted since early childhood, with an average growth velocity of 5 cm per year. He was born full term to nonconsanguineous parents via normal vaginal delivery (G2P2), with a birth weight of 3.25 kg and length of 50 cm. He experienced neonatal jaundice but recovered without complications. Developmental milestones were mildly delayed: sitting at 8 months, walking independently at 24 months, first words at 36 months, and teething at 12 months. The family history revealed an older brother, aged 11 years old with a height of 137 cm, with no developmental, neurological, or dysmorphic features and no other reported medical concerns. The father’s height is 168 cm and the mother’s 153 cm. At admission, the patient’s height was 107.4 cm (<3rd percentile, −2.74 SD) and weight was 20 kg (10–25th percentile), with a BMI of 17.47 kg/m^2^ (88th percentile for age and sex) and considered overweight (Figure 1A,B). Testicular volumes were approximately 1 mL bilaterally and penis length was 3 cm.

Laboratory tests for thyroid, adrenal, liver, and renal function were within normal ranges. Fasting glucose and homeostatic model assessment of insulin resistance (HOMA-IR, 0.97) were within normal limits, with no evidence of insulin resistance. HbA1c was 5.8%, which falls within the prediabetes range. Hormonal testing revealed a typical prepubertal profile with low gonadotropins and sex steroids, with a LH/FSH ratio of 0.07, as well as limited adrenal androgen output and normal adrenal function. IGF-1 was 127 ng/mL within the laboratory reference range (119 ± 45 ng/mL), corresponding to an approximate standard deviation score (SDS) of +0.18. Peak growth hormone after stimulation was 30.20 ng/mL. These findings do not suggest abnormalities in the growth hormone IGF-1 axis. Anti-Müllerian hormone (AMH) levels were possibly normal for the prepubertal stage but will require monitoring as sexual development progresses. Echocardiography with tissue Doppler and ultrasonography of the liver, kidneys, thyroid, and scrotum, including testicular positioning, revealed no major abnormalities. Standing full-spine radiographs showed mild lumbar lateral bending. Brain MRI was normal. The patient’s current academic performance is within the passing range for his grade level. Laboratory findings are summarized in Table 1.

### 3.2. Genetic Sequencing and Bioinformatics Investigations

**Figure 2:**
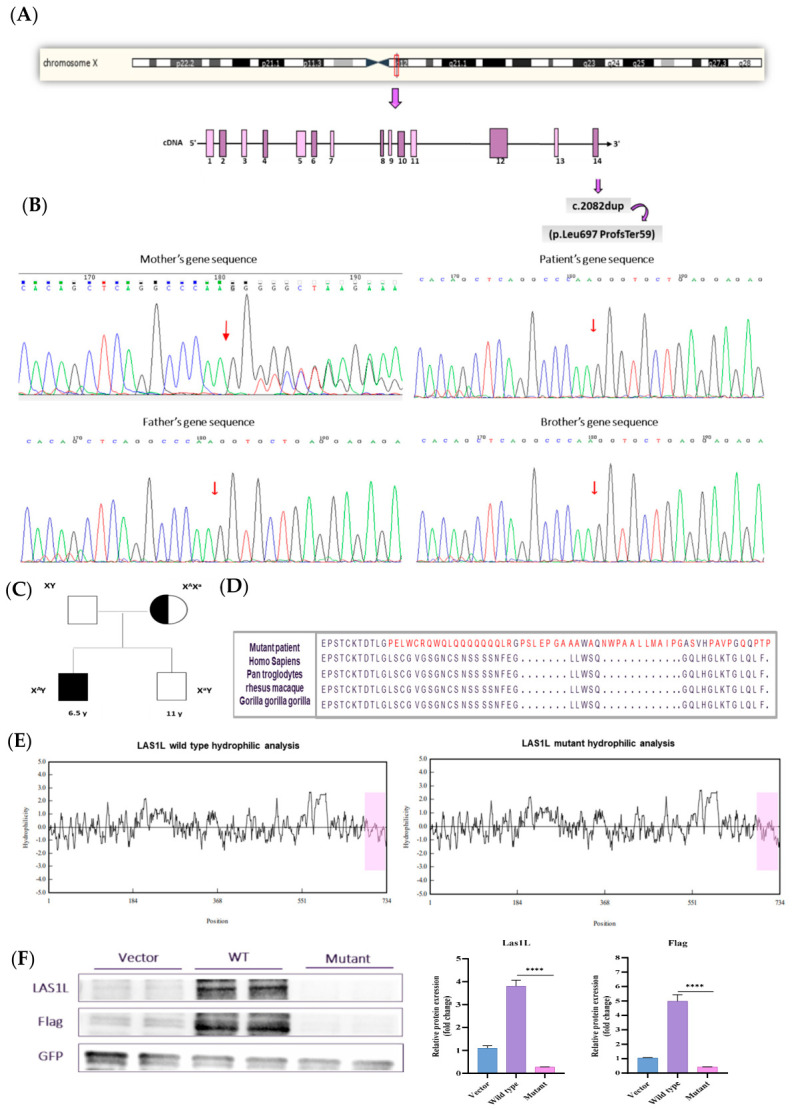
The patient’s pedigree, gene sequencing results, and functional analysis of *LAS1L* variants. (**A**) Schematic diagram of *LAS1L* cDNA structure showing the location of the variant identified in this study (The red box in panel (**A**) indicates the chromosomal region containing *LAS1L*). (**B**) Sanger sequencing results showing the *LAS1L* variant in the proband and his family. The red arrow indicates the site of the novel frameshift variant, c.2082dup (p.Leu697ProfsTer59), which consists of a single guanine (G) duplication. (**C**) Pedigree of the family in our study. (**D**) Evolutionary conservation analysis of the affected amino acid in *LAS1L*. (**E**) Hydrophobicity analysis comparing wild-type and mutant LAS1L proteins, with the altered region indicated (pink box) having potential local changes in physicochemical properties. (**F**) Representative Western blot images of FLAG-tagged *LAS1L*, endogenous *LAS1L*, and GFP in HEK-293T cells transfected with an empty vector (vector), wild-type construct (WT), or mutant construct. GFP was used as a transfection control. These results are presented descriptively as preliminary observations. **** *p* < 0.0001. Full-length uncropped blots are provided in Supplementary Figure S1.

Whole-exome sequencing identified a novel hemizygous frameshift variant in the *LAS1L* gene, c.2082dup (p.Leu697ProfsTer59), located in exon 14 (Table 2) (Figure 2A). Sanger sequencing confirmed the presence of the variant in the patient and his mother, who was a carrier, while the father and brother did not carry the variant (Figure 2B,C). Multiple sequence alignment showed that the affected region is highly conserved across vertebrates, which could have potential functional relevance (Figure 2D). The variant was not identified in population databases, including gnomAD (v4.1.0) (allele count = 0) the 1000 Genomes Project, ExAC, and dbSNP, supporting its rarity. The variant has been submitted to ClinVar (accession number VCV004759342.1). Hydrophobicity analysis showed differences in the C-terminal region between the mutant and wild-type proteins, suggesting a potential change in local protein properties; however, the functional significance of this finding remains unclear (Figure 2E).

Given the patient’s relatively mild phenotype, as well as the predicted escape of the c.2082dup variant from nonsense-mediated mRNA decay resulting in a C-terminally altered protein rather than complete loss of expression, functional analysis was performed to further characterize the variant’s potential functional impact. Western blot analysis was conducted in HEK-293T cells transfected with FLAG-tagged wild-type or mutant constructs alongside a GFP control plasmid. The wild-type construct with *LAS1L* staining showed a similar pattern while comparable GFP levels suggested similar transfection efficiency (Figure 2F). There is also a possible reduction in protein expression. However, these findings should be interpreted with caution and are presented as preliminary observations rather than definitive evidence of functional impairment.

## 4. Literature Review

To summarize the clinical manifestations reported in patients with *LAS1L* variants, available data were reviewed from HGMD and PubMed. Given the limited number of reported cases, current evidence is insufficient to establish a definitive relationship between *LAS1L* variants and a consistent WTS phenotype. Reported cases show heterogeneity in both genotype and clinical presentations, thus the following summary serves for descriptive comparison rather than causal inference. Variants in *LAS1L* have been linked to a wide spectrum of clinical phenotypes. Early studies described individuals with X-linked intellectual disability accompanied by truncal obesity and gynecomastia [4]. In contrast, multiple subsequent reports documented phenotypes distinct from classical WTS. For instance, a male neonate carrying the missense variant p.Ser477Asn presented with severe infantile motor neuron disease, hypotonia, respiratory failure, and impaired ribosome biogenesis [11]. Likewise, the splice-altering variant c.846G>C was identified in an 18-month-old boy with axonal neuropathy, hypotonia, feeding difficulties, and progressive respiratory compromise [12]. A 7-year-old male patient carrying the *LAS1L* missense variant c.1237G>A (p.Gly413Arg), with an Angelman-like neurodevelopmental phenotype including severe intellectual disability, absent speech, a happy demeanor, hyperactivity, stereotyped behaviors, ataxia, seizures, and hypotonia, was also reported, further expanding the diverse clinical spectrum of LAS1L-related disorders [6]. These observations highlight substantial phenotypic variability among individuals carrying *LAS1L* variants.

The splice site variant c.846G>C resides near an exon–intron boundary and could theoretically interfere with normal splicing, such as intron retention. However, in silico analyses from the published literature do not predict a major impact on splicing activity. *LAS1L* encodes a conserved nucleolar factor within the Rixosome complex, acting alongside PELP1, TEX10, WDR18, and NOL9 to regulate internal transcribed spacer 2 (ITS2) processing during 60S ribosomal subunit assembly [8,9,14]. Functional studies have confirmed that *LAS1L* interacts with NOL9; disruption of this interaction in model systems leads to ribosomal stress and p53 activation [7,15]. Recent work also indicates that post-translational modification of *LAS1L*, including USP36-mediated SUMOylation, modulates its function in rRNA maturation [16].

Across the five reported cases, detailed clinical phenotypes were available for only two individuals, both of whom presented with severe early-onset neuromuscular disorders. The missense variant p.Ser477Asn, reported by Butterfield et al., was found in a patient with lethal congenital motor neuron disease, characterized by profound neonatal hypotonia, ventilator-dependent respiratory failure, feeding difficulties, and death at approximately two months of age. Similarly, the splice-affecting variant c.846G>C (p.Thr282=) is associated with a SMARD-like phenotype, including early infantile hypotonia, respiratory distress, recurrent pulmonary infections, and feeding impairment [12]. While *LAS1L* variants are clearly associated with severe neuromuscular disease in these patients, the small case number prevents definitive conclusions. Two additional missense variants, p.Ala269Gly and p.Arg415Trp, were reported in classic WTS cases but lack detailed individual clinical information, which hinders robust genotype–phenotype correlation [2]. Overall, many reported cases present with neuromuscular involvement, yet interpretation remains constrained by the small sample size and incomplete phenotypic documentation.

Classic WTS features, including obesity and gynecomastia, were absent in patients with *LAS1L* variants causing infantile motor neuron disease. Different *LAS1L* variant types appear to correlate with distinct clinical presentations, though this association has not been fully validated. It has been hypothesized that variants causing severe protein dysfunction tend to result in early-onset motor neuron disease, while other variants may lead to milder or unrelated clinical manifestations [11,12]. Still, all such interpretations require caution due to the limited number of published cases. These findings align with broader research showing that impaired ribosome biogenesis can affect multiple organ systems, with clinical outcomes determined by the severity of functional disruption [17]. Cumulatively, existing data support the existence of a continuous phenotypic spectrum across LAS1L-related disorders, but this hypothesis requires validation in larger patient cohorts. An overview of variant type, predicted protein alteration, and variant location for each reported case is presented in Table 3.

## 5. Discussion

Here, we report a novel hemizygous *LAS1L* frameshift variant (c.2082dup; p.Leu697ProfsTer59) identified in a 6-year-and-5-month-old boy presenting with growth retardation, early developmental delay, and mild scoliosis. This case expands the spectrum of pathogenic *LAS1L* variants and highlights the gene’s broad phenotypic heterogeneity. The patient lacks craniofacial dysmorphism and behavioral anomalies typical of classic WTS. The identified C-terminal frameshift variant is biologically consistent with the patient’s growth and developmental and skeletal phenotypes. Its absence from general population databases, compatibility with X-linked inheritance, and evidence of reduced mutant protein expression support classification of c.2082dup as likely pathogenic, though further functional validation is warranted.

Accumulating evidence indicates that LAS1L-related phenotypes extend beyond classic WTS, encompassing mild–intermediate presentations with minimal endocrine or metabolic involvement. The identified single nucleotide duplication induces a frameshift that disrupts the protein’s C-terminal domain. As the frameshift variant is located in the final exon, the mutant transcript is predicted to escape NMD. Therefore, this variant may result in the production of a LAS1L protein with an altered C-terminal sequence instead of causing complete loss of LAS1L function [18]. In silico hydrophobicity analysis suggested altered local protein physicochemical properties, and the variant is extremely rare in public population databases.

In accordance with ACMG/AMP 2015 guidelines, the variant was classified as likely pathogenic, supported by PVS1_Moderate evidence for disruptive frameshift alteration of a conserved domain, PS3_Supporting evidence for impaired protein expression in vitro, and PM2_Supporting evidence for population rarity [19]. Familial testing confirmed consistent X-linked inheritance, as the variant was present in the proband but absent in his unaffected sibling. In vitro assays in HEK-293T cells verified the reduced expression of mutant LAS1L protein.

Two well-defined severe phenotypic patterns characterize LAS1L-related disease: classic WTS, which presents with X-linked intellectual disability, truncal obesity, hypogonadism, gynecomastia, and speech delay [2]; and a lethal infantile neuromuscular disorder, marked by severe hypotonia, respiratory failure, joint contractures, and early mortality, as well as phenotypic overlap with SMARD [11,12]. The present patient’s clinical profile does not match either severe subtype, with no history of neonatal decompensation, severe hypotonia, or motor neuron disease.

As a prepubertal child, the patient exhibits no classic WTS endocrine or dysmorphic features. He demonstrates mild metabolic dysregulation, with overweight BMI, elevated HbA1c, and increased 1 h postprandial glucose, suggestive of early glucose intolerance. Consistent with the expanding *LAS1L* disease spectrum, his combination of growth impairment, early developmental delay, and mild scoliosis represents a mild–intermediate phenotype. Since hallmark WTS metabolic and pubertal features typically emerge in late childhood and adolescence, the patient’s clinical presentation may evolve over time. The retention of partial protein function due to escape from nonsense-mediated decay likely contributes to his attenuated disease severity.

The wide phenotypic variability associated with *LAS1L* variants challenges traditional WTS diagnostic criteria. Identification of a pathogenic or likely pathogenic *LAS1L* variant alone is not sufficient for a definitive WTS diagnosis, given this patient’s unique non-classic presentation. Longitudinal pubertal follow-up and additional case reports will clarify whether such mild presentations represent age-dependent WTS or distinct non-classic *LAS1L* phenotypes.

This phenotypic heterogeneity raises important questions regarding WTS diagnostic boundaries. Including the present case, six LAS1L-related molecular observations have been reported to date, strongly supporting a spectrum-based classification model for LAS1L-related disorders.

This study can provide practical implications for clinical diagnosis and management. LAS1L-related disorders could be included in the differential diagnosis of children with unexplained growth failure, developmental delay, and early scoliosis, even in the absence of typical WTS features. Clinical exome sequencing facilitates molecular diagnosis in such undiagnosed syndromic cases, and functional protein assays support variant interpretation. Due to the variable endocrine and metabolic expressivity of *LAS1L* variants, affected individuals require baseline and longitudinal endocrinological surveillance covering growth, glucose homeostasis, thyroid function, gonadal function, and adrenal function. Pituitary imaging may be implemented clinically to evaluate potential hypothalamic-pituitary dysfunction.

Structured long-term monitoring is essential for this prepubertal patient to track potential disease progression. Regular serial assessment of body composition and glycemic metabolism is recommended alongside annual endocrine surveillance, with focused hypothalamic–pituitary–gonadal axis evaluation starting in pre-adolescence to screen for pubertal-onset WTS features. Orthopedic follow-up is required to monitor scoliosis progression, while annual developmental and behavioral assessments track long-term functional outcomes.

This single case study has inherent limitations. It cannot fully define the phenotypic spectrum of LAS1L-related disease or establish definitive genotype–phenotype correlations. Furthermore, in vitro functional assays do not completely recapitulate human in vivo pathophysiology. Larger cohort studies are necessary to refine genotype–phenotype relationships and characterize the natural history of LAS1L-associated disorders.

Overall, this partial loss of function *LAS1L* variant illustrates possible residual ribosomal protein function drives intermediate, mild phenotypes within the ribosomopathy spectrum and emphasize the necessity of individualized clinical assessment and long-term multidisciplinary monitoring for all patients with LAS1L-related disorders, including non-classic presentations of LAS1L-related WTS.

## 6. Conclusions

In conclusion, we report a patient presenting with growth retardation, early developmental delay, and mild scoliosis harboring a novel *LAS1L* frameshift variant, expanding the limited spectrum of published LAS1L-related cases. This case further demonstrates the broad clinical variability associated with *LAS1L* variants and confirms that LAS1L-related disease can present as mild–intermediate phenotypes without classical WTS features. For patients with unexplained growth failure and developmental delay and a suspected genetic cause, *LAS1L* could be included in the differential diagnosis. This study also emphasizes the value of long-term longitudinal follow-up to characterize the potential evolution of clinical features throughout childhood and puberty. Further investigations are necessary to refine genotype–phenotype correlations and fully define the clinical spectrum of LAS1L-related disorders.

## Figures and Tables

**Table 1:** Summary of laboratory findings.

Parameter	Value	Reference
Fasting glucose	5.2 mmol/L	3.9–6.1 mmol/L
HOMA-IR	0.97	<2.5
HbA1c	5.8%	4.0–5.7%
Postprandial glucose (1 h)	9.26 mmol/L	<9.0
Insulin (1 h)	56.4 µIU/mL	5–10 × fasting insulin
Insulin (2 h)	36 µIU/mL	2–4 × fasting insulin
Basal LH	0.67 mIU/mL	0.1–0.8 mIU/mL
Basal FSH	10 mIU/mL	0.3–10.0 mIU/mL
Peak LH	2.61 mIU/mL	<5.0 mIU/mL
Peak FSH	4.90 mIU/mL	<10.0 mIU/mL
IGF1	127 ng/mL	119 ± 45 ng/mL
Peak GH	30.20 ng/mL	≥10 ng/mL
Testosterone	<0.01 nmol/L	<0.5 nmol/L
Estradiol	10 pg/mL	<15 pg/mL
ACTH	18.1 pg/mL	25–100 pg/mL
Cortisol	236 nmol/L	135–650 nmol/L
DHEA-S	489 nmol/L	384 ± 61 nmol/L
Androstenedione	<0.01 nmol/L	0.71 ± 0.4 nmol/L

**Table 2:** Genetic details of the *LAS1L* variant identified in the patient.

Gene	Chromosomal Position	Transcript Exon	Nucleotide Amino Acids	Zygosity	Disease/Phenotype (Mode of Inheritance)
*LAS1L*	chrX:64732777-64732778	NM_031206.7;exon 14	c.2082dup (p.Leu697ProfsTer59)	Hemizygous	LAS1L-related disorder (X-linked; inherited from mother)

**Table 3:** Genotype table outlining each patient’s variant type, predicted protein alteration, and location within the protein.

Gender	Age	cDNA Variant	Variant Class	Protein Alteration	Domain Location	Reference
Male	Newborn infant, died ~2 months	c.1430G>A	Missense	p.Ser477Asn	Conserved internal region (likely functional domain)	[11]
Male	18 months old	c.846G>C	Splice affecting	p.Thr282=	N-terminal region, exon 6	[12]
Male	Not specified	Not specified	Missense	p.Ala269Gly	N-terminal region (domain not well characterized)	[2]
Male	Not specified	Not specified	Missense	p.Arg415Trp	Central region (conserved functional region)	[2]
Male	7 years	c.1237G>A	Missense	p.Gly413Arg	Central region	[6]
Male	6.5 years old	c.2082dup	Frameshift	p.Leu697ProfsTer59	C-terminal, downstream of HEAT repeats	Present case

## Data Availability

The data that support the findings of this study are available from the corresponding author upon reasonable request. The variant identified in this study has been deposited in the ClinVar database under accession number VCV004759342.1.

## Supplementary Materials

The following supporting information can be downloaded at: https://www.mdpi.com/article/10.3390/genes17060708/s1, Figure S1: Full-length original blots; Table S1: Primer sequences designed and used in this study; Table S2: Primary antibodies used in this study.

## Abbreviations

WTS	Wilson–Turner Syndrome
*LAS1L*	LAS1-Like Ribosome Biogenesis Factor
WES	Whole-Exome Sequencing
PCR	Polymerase Chain Reaction
HGMD	Human Gene Mutation Database
OMIM	Online Mendelian Inheritance in Man
ACMG/AMP	American College of Medical Genetics and Genomics/Association for Molecular Pathology
HEK-293T	Human Embryonic Kidney 293T Cell
WT	Wild Type
RIPA	Radioimmunoprecipitation Assay
DMEM	Dulbecco’s Modified Eagle Medium
LH	Luteinizing Hormone
FSH	Follicle-Stimulating Hormone
GH	Growth Hormone
ACTH	Adrenocorticotropic Hormone
DHEA-S	Dehydroepiandrosterone Sulfate
OGTT	Oral Glucose Tolerance Test
AMH	Anti-Müllerian Hormone
HOMA-IR	Homeostatic Model Assessment of Insulin Resistance
BMI	Body Mass Index
MRI	Magnetic Resonance Imaging
SEM	Standard Error of Mean
NMD	Nonsense-Mediated mRNA Decay
SMARD	Spinal Muscular Atrophy with Respiratory Distress
