# Supplementary material for: A Novel LAS1L Gene Mutation Associated with Impaired Growth and Developmental Delay and a Review with Previously Reported Cases
Source: Genes (Basel). 2026 Jun 20;17(6):708. doi: 10.3390/genes17060708 (PMC13299373; doi:10.3390/genes17060708)

Table S1. Primer sequences designed and used in this study

**Gene**	Forward primer	Reverse primer
LAS1L	AGGTTGTCTCAGGGAGCATC	TAAAGCTGCCTCC+

Table S2. Primary antibodies used in this study

Primary antibodies	Company	Article number
LAS1L Rabblit pAb	Proteintech	16010-1-AP
Flag Rabblit pAb	Abclonal	AE092
GFP Rabblit pAb	CST	2956

Figure S1. Full-length original blots

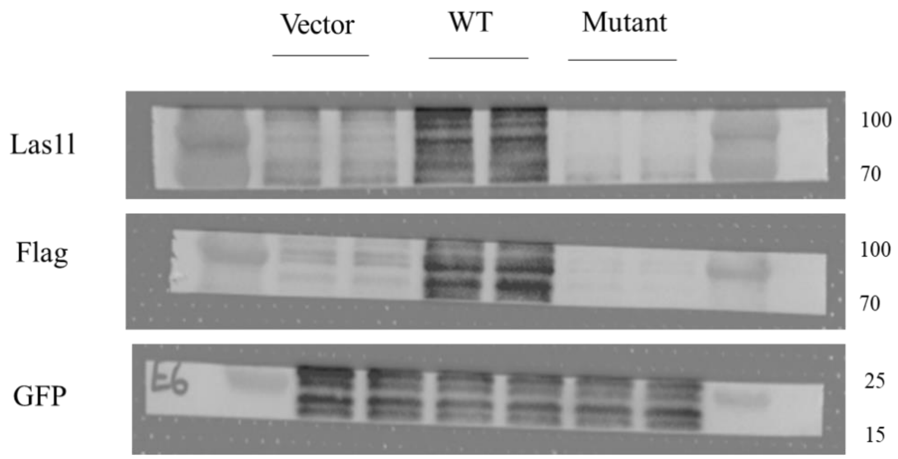

Supplement: Supplementary file 1 [file genes-17-00708-s001.zip › genes-4346898-supplementary.pdf]
